# Molecular mechanism mediating enteric bacterial translocation after severe burn: the role of cystic fibrosis transmembrane conductance regulator

**DOI:** 10.1093/burnst/tkaa042

**Published:** 2021-01-15

**Authors:** Xinzhu Liu, Yu Chen, Bo You, Yuan Peng, Yajie Chen, Zichen Yang, Yixin Zhang, Jing Chen

**Affiliations:** 1 State Key Laboratory of Trauma, Burns and Combined Injury, Chongqing Key Laboratory for Proteomics Disease, Institute of Burn Research, Southwest Hospital (the First Affiliated Hospital), Third Military Medical University (Army Military Medical University), Gao Tan Yan Street, Chongqing 400038, China; 2 Department of Burn and Plastic Surgery, No. 958 Hospital of Army, Southwest Hospital, Third Military Medical University (Army Military Medical University), Jian Xin Dong Street, Chongqing 400020, China; 3 Department of Plastic and Reconstructive Surgery, Shanghai Ninth People’s Hospital, Shanghai Jiao Tong University School of Medicine, Zhi Zao Ju Road, Shanghai 200011, China

**Keywords:** Burn, Inflammation, Intestinal junction, Enteric bacterial translocation, Vitamin D3, Bacterial translocation, Cystic fibrosis transmembrane

## Abstract

**Background:**

Gut ischemia and hypoxia post severe burn leads to breakdown of intestinal epithelial barrier and enteric bacterial translocation (EBT), resulting in serious complications, such as systemic inflammatory response syndrome, sepsis and multiple organ failure. Cystic fibrosis transmembrane conductance regulator (CFTR) is known to be downregulated by hypoxia and modulate junctional complexes, which are crucial structures maintaining the intestinal barrier. This study aimed to investigate whether CFTR plays a role in both regulating the intestinal barrier and mediating EBT post severe burn, as well as the signaling pathways involved in these processes.

**Methods:**

An *in vitro* Caco-2 cell model subjected to hypoxic injury and an *in vivo* mouse model with a 30% total body surface area full-thickness dermal burn were established. DF 508 mice (mice with F508del CFTR gene mutation) were used as an in vivo model to further demonstrate the role of CFTR in maintaining normal intestinal barrier function. QRT-PCR, western blot, ELISA, TER assay and immunofluorescence staining were used to detect the expression and localization of CFTR and tight junction proteins, as well as the function of tight junctions.

**Results:**

Our data indicated that, in Caco-2 cells, the hypoxia condition significantly reduced CFTR expression; activated extracellular signal-regulated kinase and nuclear factor-κB signaling; elevated secretion of inflammatory factors (tumor necrosis factor-α, interleukin-1β and interleukin-8); downregulated zonula occludens-1, occludin and E-cadherin expression; decreased transepithelial electrical resistance values; and led to a cellular mislocation of ZO-1. More importantly, knockdown of CFTR caused similar alterations. The upregulation of inflammatory factors and downregulation of tight junction proteins (ZO-1 and occludin) induced by knockdown of CFTR could be reversed by specific extracellular signal-regulated kinase or nuclear factor-κB inhibition. In support of the *in vitro* data, exuberant secretion of pro-inflammatory mediators and EBT was observed in the intestine of severely burnt mice *in vivo*. EBT occurred in DF508 mice (mice with the F508del CFTR gene mutation), accompanied by augmented tumor necrosis factor-α, interleukin-1β and interleukin-8 levels in the ileum compared to wildtype mice. In addition, vitamin D3 was shown to protect the intestinal epithelial barrier from hypoxic injury.

**Conclusions:**

Collectively, the present study illustrated that CFTR and downstream signaling were critical in modulating the intestinal epithelial junction and EBT post severe burn.

Highlights

- This study revealed a crucial role of CFTR in regulating intestinal tight junctions, thus modulating enteric bacterial translocation after severe burn injury.
- We also demonstrated an extracellular signal-regulated kinase/nuclear factor-κB and inflammatory signaling pathway downstream of CFTR, mediating intestinal barrier disruption after severe burn.
- Vitamin D3 protected the intestinal barrier from serious burn injury both *in vitro* and *in vivo*, thus shedding new light on the application of vitamin D3 in severely burnt patients.

## Background

After severe burn, organ blood flow is redistributed in favor of vital organs, such as the heart and brain, and therefore blood flow to the gut is markedly decreased. Intestinal ischemia and hypoxia cause disturbance of the intestinal epithelial barrier, followed by enteric bacterial translocation (EBT) [1], contributing greatly to the development of systemic inflammatory response syndrome, sepsis and Multiple Organ Dysfunction Syndrome, which are the common causes of mortality in major burn injury [2–8]. Intestinal epithelial layers are tightly linked by apical intercellular junctional complexes, comprised of tight junctions (TJs), adherens junctions (AJs) and desmosome-like junctions, segregating commensal bacteria in the intestinal lumen from systemic circulation [9]. zonula occludens-1 (ZO-1) and occludin, in particular, are key elements in the structure of TJ complexes. The AJ protein E-cadherin mediates cell–cell adhesions, which are crucial for the assembly and stability of TJs [10]. Once junctional complexes are compromised, EBT takes place [11]. Though EBT post severe burn has been widely investigated, the underlying molecular mechanisms remain poorly understood.

Cystic fibrosis transmembrane conductance regulator (CFTR) is an ion channel, widely expressed in epithelial cells, mutation (mostly at position 508, DF508) of which causes a genetic disorder, cystic fibrosis (CF) that affects the lung, pancreas, liver, kidneys and intestine [12]. CF is characterized by severe airway infection. A previous study indicated that lung-colonizing microbes in CF animal models may be translocated from enteric bacteria [13–15], suggesting a potential role of the CFTR defect in EBT. Moreover, it is well known that the function and expression of CFTR is suppressed by hypoxia in a number of cells and tissues, including intestinal epithelium [16, 17]. Thus, CFTR impairment induced by enteric hypoxia may play a part in EBT post severe burn.

Increased gut mucosal permeability and decreased expression of junctional complex proteins in the intestinal epithelia have been observed after major burn injury [18–20]. Interestingly, mutations of CFTR, which lead to defect of the protein leads to similar intestinal alterations. Permeability is increased in the intestines of patients with CF and CF mice [21]. It has been demonstrated previously that CFTR can regulate junctional complexes proteins [22]. One of the underlying mechanisms is the excessive inflammation observed in the CF small intestine, as revealed by wireless capsule endoscopic results [23]. Knockdown of CFTR in intestinal epithelia results in an augmented release of pro-inflammatory cytokines, including interleukin-1β (IL-1β) and interleukin-8 (IL-8) [24]. Moreover, tumor necrosis factor-α (TNF-α), IL-1β and IL-8 are known to disrupt TJs and induce intestinal barrier defects [25–27]. Intriguingly, these cytokines are also dramatically elevated in the gut of severely burnt mice, rats and humans [28–30].

Therefore, it is reasonable to speculate that CFTR and the increased release of cytokines may be involved in gut barrier disruption post severe burn. Furthermore, nuclear factor-κB (NF-κB) and mitogen-activated protein kinase (MAPK) signaling are regarded as pathways mediating cytokine production regulated by CFTR, and our previous study has identified CFTR-regulated MAPK/NF-κB signaling in pulmonary epithelial cells after burn [31–33]. Therefore, MAPK/NF-κB signaling may be also involved in this process.

A number of studies have illustrated that vitamin D3 exerts protective effects on intestinal epithelial barrier and prevents EBT [34, 35]. One of the pivotal mechanisms involves the anti-inflammatory action of vitamin D3 on the intestinal epithelia [36]. Thus, application of vitamin D3 to severely burnt mice may ameliorate inflammation and protect the junctional complexes of the intestine.

Collectively, the present study aims to demonstrate that, following severe cutaneous burn injury, CFTR plays an important role in preventing EBT by inhibiting inflammation and maintaining an intact intestinal epithelial barrier. This study also intends to test whether administration of vitamin D3 is beneficial to the intestine post severe burn.

## Methods

### Cell culture and treatments

Caco-2, a human adenocarcinoma cell line widely used in studies on EBT, was employed in this study. Caco-2 cells (ATCC), China) were cultured in Eagle’s Minimum Essential Medium (ATCC) with 20% fetal bovine serum in a CO_2_ (5%) incubator at 37°C. When the monolayer was prepared, Caco-2 cells were plated at a density of 1 × 10^5^ cells/cm^2^ and grown on a collagen-precoated permeable polycarbonate membrane transwell consisting of 0.4-μm pores (Corning, USA) and used for subsequent experiments after full confluence. For treatment of hypoxia, Caco-2 monolayers were exposed to 5% CO_2_, 1% O_2_ and 94% N_2_ for 6 or 12 hours. For the vitamin D3 treatment group, 1,25-dihydroxyvitamin D3 (#740543, Sigma, USA) was dissolved in a solvent composed of distilled deionized water, propylene glycol and ethanol (volume ratio 5:4:1). Cells were treated with a final concentration of 10^−7^ M 1,25-dihydroxyvitamin D3, or solvent as vehicle control, concomitant with exposure to the hypoxic condition. Vehicle or vitamin D3 was added to the basolateral compartment of the Caco-2 monolayer setup. A specific ERK signaling inhibitor, PD98059 (Sigma, USA) and a specific NF-κB inhibitor, Bay 11-7082 (Sigma, USA) were used in this study.

### CFTR knockdown

CFTR was knocked down by transfection of 4 μg hammerhead ribozymes (conjugated in pEF6/V5-His vector) targeting a specific GUC or AUC site to degrade CFTR mRNA, with an empty pEF6/V5-His vector used as control. Lipofectamine 2000 reagent (Invitrogen, USA) was used for transfection according to the manufacturer’s instructions.

### Transepithelial electrical resistance assay

Caco-2 cells were cultured on collagen-coated transwell polycarbonate membrane filter inserts (Corning). The cells were seeded at a density of 1 × 10^5^ cells/ml. The medium was changed every 1 or 2 days. The integrity of the monolayer was closely observed after culturing for approximately 12 days and evaluated by measuring its transepithelial electrical resistance (TER), using the Millicell Electrical Resistance System (Millipore). All TER values were normalized to the control group.

### Animals and procedures

The DF508 mice were purchased from the Jackson Laboratory, USA. Male adult DF508 mice were used for the experiments Si Pei Fu Biotechnology, China. Male C57BL/6 mice were used to establish the burn model. All procedures were approved by the Animal Ethical Committee of the Third Military Medical University and were carried out according to the approved guidelines of the animal center of Southwest Hospital, Third Military Medical University.

To evaluate the effect of vitamin D3 on EBT post burn, C57BL/6 mice were randomized into one of 3 groups (sham, burn and vehicle, burn and vitamin D3) and anesthetized via intraperitoneal (i.p.) injection with a mixture of ketamine (75 mg/kg) and xylazine (10 mg/kg). A 30% total body surface area (TBSA) full-thickness dermal burn model was established as follows. After removing dorsal hairs, the mouse was placed in an insulating mold device with an opening exposing 30% TBSA and immersed in 62°C water for 25 seconds, as previously described [37]. All mice received sterile saline (50 ml/kg i.p.) for fluid resuscitation immediately after burn or sham treatment. Buprenorphine (0.1 mg/kg) was given by subcutaneous injection to all mice for analgesia immediately after the surgery. In the vitamin D3 treatment group, at 48 hours, 24 hours and 1 hour before burn treatment, mice were given either vitamin D3, at a dose of 100 ng/kg body weight, or vehicle control by i.p. injection. Distal ileum was removed at 6, 12 or 24 hours post burn for further experiments, since EBT is prone to occur in this segment of intestine. Lymph nodes, spleen and liver of mice were excised for analysis for bacterial counts.

### Analysis of bacterial counts in extraintestinal organs

The mesentery lymph nodes, spleens and livers of mice were excised and weighed. Tissues were homogenized and sonicated in sterile phosphate-buffered saline (PBS) at a concentration of 0.1 g/ml. The tissue homogenates (200 μl per plate) were cultured at 37°C overnight on fresh blood agar plates (Pang Tong Medical Devices Co., Ltd, China). The number of bacterial colony-forming units was normalized to per gram of tissue.

### Real-time quantitative RT-PCR

Real-time quantitative PCR (QRT-PCR) was carried out using a complementary DNA synthesis kit (Thermo Scientific, USA) and the PCR mix (SGExcel UltraSYBR Mixture, Sangon Biotech, China). A 96-well plate was used for the PCRs. Assays were performed in triplicate on a Real Time PCR System (CFX96 Real-Time System, Bio-rad, USA) and average cycle threshold (Ct) values were normalized relative to the expression of β-actin. The sequences of primers used are listed in Table 1.

**Table 1:** Sequences of primers used in experiments

Human cystic fibrosis transmembrane conductance regulator	TGCCCTTCGGCGATGTTT (forward)
	GCGATAGAGCGTTCCTCCTTG (reverse)
Human zonula occludens-1	CAACATACAGTGACGCTTCACA (forward)
	CACTATTGACGTTTCCCCACTC (reverse)
Human occludin	GACTTCAGGCAGCCTCGTTAC (forward)
	GCCAGTTGTGTAGTCTGTCTCA (reverse)
Human interleukin-1β	GCATCCAGCTACGAATCTCC (forward)
	AGGGAACCAGCATCTTCCTC (reverse)
Human interleukin-8	ACTGAGAGTGATTGAGAGTGGAC (forward)
	AACCCTCTGCACCCAGTTTTC (reverse)
Human tumor necrosis factor-α	TCTGGGCAGGTCTACTTTGG (forward)
	GGTTGAGGGTGTCTGAAGGA (reverse)
Human β-actin	CATGTACGTTGCTATCCAGGC (forward)
	CTCCTTAATGTCACGCACGAT (reverse)

### Immunofluorescence staining

Segments of distal ileum (n = 3 animals per group) were embedded in optimum cutting temperature compound OCT media. Sections of intestine were cut (10 μm thick) at −20°C and fixed onto glass slides with cold acetone, washed with PBS and then permeabilized with 1% Triton X-100 (Sigma, USA). After blocking in 3% bovine serum albumin for 1 hour, the sections were then incubated overnight in CFTR (1:100; Alomone Labs, Israel) and ZO-1 (1:50; Proteintech, USA) antibodies. They were then treated with FITC-conjugated Affinipure goat anti-rabbit (1:50; proteintech, USA) or TRITC-conjugated Affinipure goat anti-rabbit (1:50; Beijing Dingguo Changsheng Biotechnology, China) antibodies in 1% BSA for 1 hour. Images were viewed using a laser-scanning confocal microscope (Zeiss LSM800, Germany). Specificity of all immunostainings was checked by incubating the tissue sections with secondary antibodies only, and no background staining was found (negative control). Five random fields were examined per animal to detect CFTR expression level. The level of CFTR and ZO-1 expression was quantified by measuring the mean pixel intensity (MPI) of CFTR or ZO-1 fluorescent labeling in the intestine.

Caco-2 cells were grown on coverslips. After confluence, cells were fixed with acetone, blocked in 10% goat serum in PBS and incubated with antibodies against ZO-1 (1:50; proteintech), occludin (1:50; proteintech) or NF-κB p65 (1:50; Cell Signaling Technology, USA) at 4°C overnight, followed by 1 hour of incubation with FITC-conjugated Affinipure goat anti-rabbit (1:50; Proteintech) or TRITC-conjugated Affinipure goat anti-rabbit (1:50; Beijing Dingguo Changsheng Biotechnology) antibodies in 1% BSA. Pictures were taken using a laser-scanning confocal microscope (Zeiss LSM800).

### Western blot

Western blot was used to detect target protein expression. The antibodies used were: CFTR (1:200; #ACL-006, Alomone Labs), extracellular signal-regulated kinase (ERK) (1:1000; #4695, Cell Signaling Technology), p-phospho-extracellular signal-regulated kinase (1:2000; #4370, Cell Signaling Technology), ZO-1(1:500; #61-7300, Thermo Scientific), occludin(1:500; #AB21068a, BBI Life Sciences, China), E-cadherin (1:500; #sc-7870, Santa Cruz Biotechnology, USA), β-actin (1:1000; #60008-1-Ig, Proteintech) and β-tubulin (1:1000; #10068-1-AP, Proteintech).

### Enzyme-linked immunosorbent assay

Enzyme-linked immunosorbent assay kits for human TNF-α (D710599), IL-1β (D710327) and IL-8 (D710724) and mouse TNF-α (D720008), IL-1β (D720335) and IL-8 (D720368) were purchased from Sangon Biotech, China and used according to manufacturer’s instructions.

### Statistical analysis

Data are shown as the mean ± SEM. Student’s *t*-test was used to compare 2 groups. One-way analysis of variance, followed by Tukey’s post hoc test, was used to compare more than 2 groups. Repeated-measures analysis of variance was used to analyse the TER values. Multivariate analysis of variance was used to compare 2 groups at each time point. Statistical significance was set at *p* < 0.05.

## Results

### CFTR, ZO-1, occludin and E-cadherin in intestinal epithelia were decreased after severe burn

**Figure 1.**
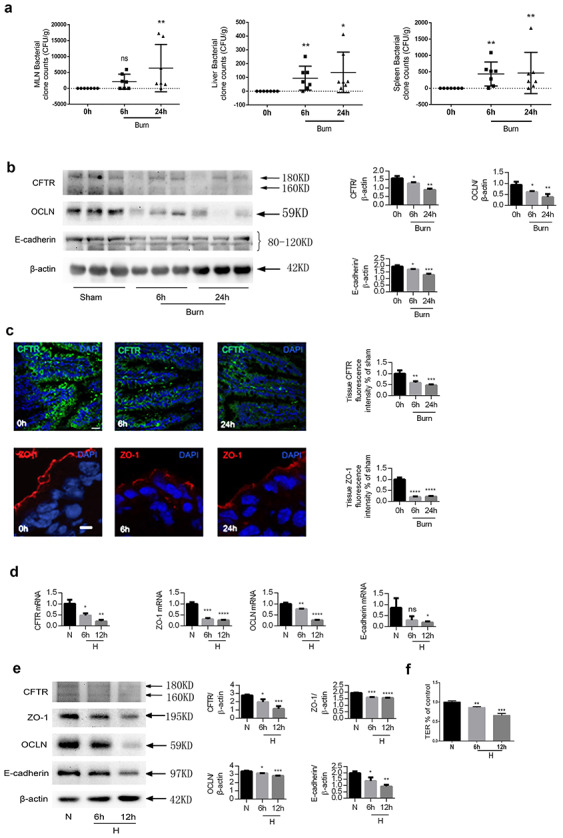
Decrease of CFTR, ZO-1, occludin and E-cadherin in intestinal epithelia post severe burn. **(a)** Bacterial counts of homogenates of lymph nodes, spleen and liver of sham or burnt mice. Data are means ± SEM from at least 3 independent experiments, n = 4. **(b)** Western blotting of CFTR, occludin, E-cadherin and β-actin of the ileum of C57BL/6 mice with (burn) or without (sham) 30% TBSA full-thickness cutaneous burn (left panel) and statistical analysis (right panel). Band B (160KD) and Band C (180KD) of CFTR showing different levels of glycosylation of CFTR are indicated by arrows. As band C (180KD) represents the fully glycosylated mature CFTR protein, which functions as an ion channel, the quantitative bar graph corresponds to band C. **(c)** Representative images and statistical analysis of immunofluorescence staining for CFTR and ZO-1 in the ilea of sham and burnt mice. Scale bar = 20 μm. To prepare the monolayer, Caco-2 cells were seeded in collagen-precoated permeable polycarbonate membrane transwell with 0.4-μm pores and used for experiments after confluence. Caco-2 monolayers were exposed to 1% O_2_ for 6 or 12 hours as hypoxia treatment or without hypoxia treatment (H, hypoxia). Real-time quantitative PCR **(d)** and western blot **(e)** analyses of CFTR, ZO-1, occludin and E-cadherin were performed. **(f)** TER measurement of Caco-2 monolayers with or without hypoxia. All TER values were normalized to the control group (normoxia). One-way ANOVA followed by Tukey’s post hoc test was used when there were more than 2 groups. The experiments were repeated at least twice. ^*^p < 0.05, ^**^p < 0.01, ^***^p < 0.001, ^****^p < 0.0001. *CFTR* cystic fibrosis transmembrane conductance regulator, *DAPI* 4′,6-Diamidino-2-phenylindole, *MLN* mesenteric lymph nodes, *N* normoxia, *OCLN* occludin, *TER* transepithelial resistance *ZO*-1 zonula occludens-1, *TBSA* total body surface area

A mouse model of cutaneous burn (30% TBSA full-thickness burn on the back) was established. The occurrence of EBT in the burnt mice was proven by bacterial isolation from the mesenteric lymph nodes, liver and spleen (Figure 1a). As the majority of previous studies in this area used the terminal ileum to study the intestinal barrier and EBT, distal ileums of the mice were resected to examine CFTR, occludin, E-cadherin and ZO-1 expression at 6 and 24 hours post burn injury. Western blot analysis showed a remarkable time-dependent decrease of CFTR, occludin and E-cadherin in the distal ileum after burn injury (Figure 1b). Immunofluorescence staining showed a markedly reduced CFTR level in the ileal epithelia at both 6 and 24 hours post burn injury. ZO-1 staining was also attenuated post burn and showed varying degrees of loss from the TJ and apico-lateral surfaces (Figure 1c).

Enteric ischemia and hypoxemia are well-known early events in the pathophysiology of burn-induced EBT [38, 39], which downregulates CFTR expression in intestinal epithelia [16, 17]. To test this hypothesis, Caco-2, an intestinal epithelial cell line widely used to study EBT, was treated with hypoxia (1% oxygen) and CFTR expression was examined. As a previous study showed that intestinal blood and oxygen supply returned to normal within 24 hours post burn, Caco-2 cells were subjected to hypoxic condition for either 6 or 12 hours. Significant, time-dependent downregulation of CFTR, accompanied by a decline of ZO-1, occludin and E-cadherin, was detected by QRT-PCR (Figure 1d) and western blot (Figure 1e). As an indicator of junctional function, TER was measured and found to be significantly reduced in the Caco-2 cell monolayers after hypoxia (Figure 1f).

### CFTR-regulated intestinal junctions

**Figure 2.**
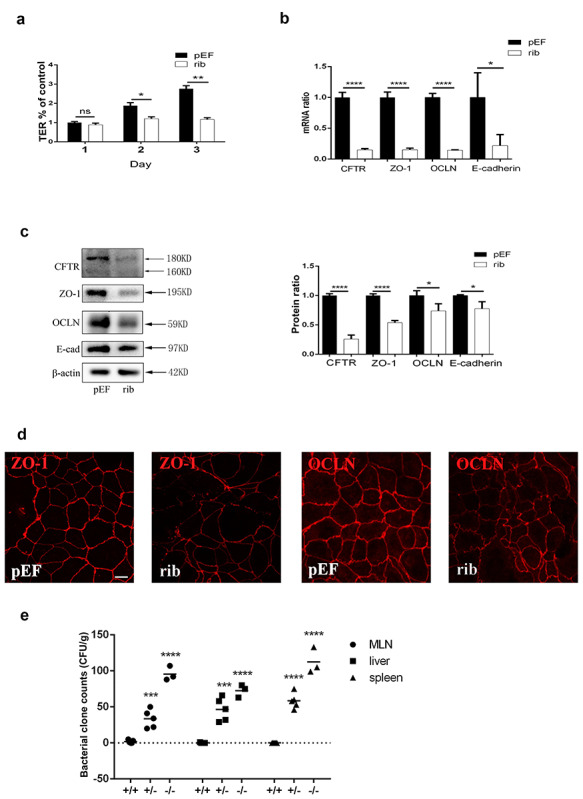
Knockdown of CFTR leads to alterations in expression and localization of tight junction proteins. Caco-2 cells were seeded onto permeable supports at a density of 2 × 10^5^ cells/ml and were transfected with CFTR-silencing ribosome vectors (**rib**) or empty pEF/V5-His vectors as negative control (**pEF**) on the second day. **(a)** TER measurement of Caco-2 monolayers on 1, 2 and 3 days post transfection. All TER values were normalized to the control group (pEF day 1). Repeated-measures analysis of variance was used to do the statistical analysis. Multivariate analysis of variance was used to compare the two groups at each time point; n = 3. **(b)** Real-time quantitative PCR and **(c)** western blot of CFTR, ZO-1, occludin and E-cadherin (left panel) and statistical analysis using unpaired *t-*test; (n = 3). **(d)** Immunofluorescence staining for ZO-1 and occludin on the third day after transfection with pEF or rib. Scale bar =10 μm. **(e)** Bacterial counts of homogenates of lymph nodes, spleen and liver of DF 508 +/+, +/− and −/− mice, and results of one-way analysis of variance using Tukey's multiple comparisons test for post hoc comparisons. For +/+, n = 5; +/−, n = 5; and −/−, n = 3. ^*^p < 0.05, ^**^p < 0.01, ^***^p < 0.001, ^****^p < 0.0001. The experiments were repeated at least twice. *CFTR* cystic fibrosis transmembrane conductance regulator, *OCLN* occludin, *TER* transepithelial resistance, *ZO*-1 zonula occludens-1

We proceeded to investigate whether CFTR could regulate intestinal epithelial junctions by knockdown of CFTR with hammerhead ribozymes degrading CFTR mRNA in Caco-2 cell monolayers. The results demonstrated that, compared to cells transfected with vehicle control vectors, cells transfected with CFTR knockdown vectors showed a remarkable decrease of TER (Figure 2a), reduced ZO-1, occludin and E-cadherin expression (Figures 2b and c), and a disorganized morphology of TJs, indicated by ZO-1 and occludin immunofluorescence staining (Figure 2d).

To determine whether CFTR played a role in EBT *in vivo*, we took advantage of CFTR-mutated DF508 mice. By comparing bacterial clone counts of mesenteric lymph node, liver and spleen homogenate cultures from DF508 mice with 3 different genotypes, we found no bacterial translocation in the wild-type (+/+) group of mice; the greatest number of translocated bacteria were found in the mutant (−/−) group of mice (Figure 2e), suggesting an important role of CFTR in EBT.

### CFTR regulated intestinal junctions through ERK, NF-κB and inflammatory factors

**Figure 3.**
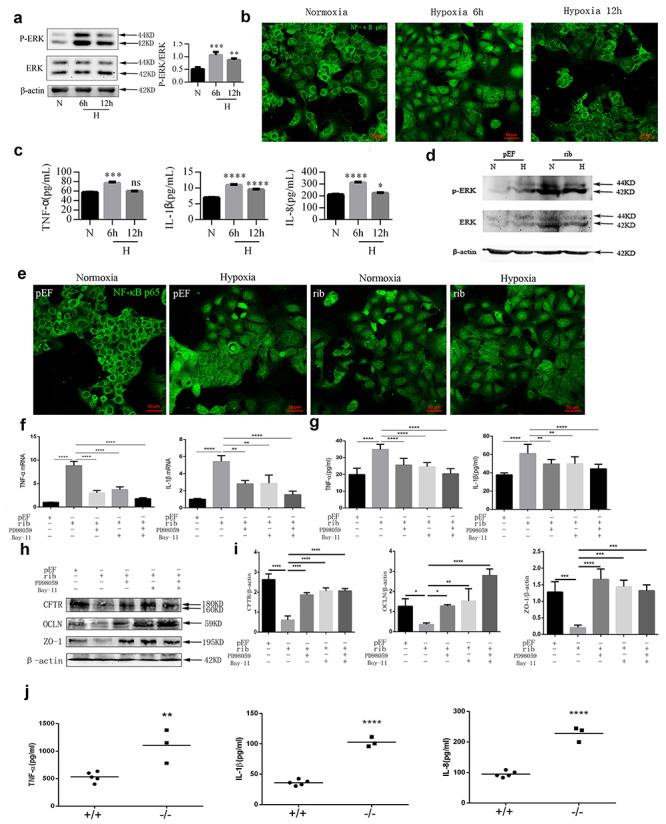
Hypoxia or knockdown of CFTR activated ERK and NF-κB signaling and promoted cytokine secretion. **(a)** Western blotting for Phospho-extracellular signal-regulated kinase (P-ERK) and ERK of Caco-2 cells treated with normoxia or hypoxia (6 or 12 hours). Results of one-way ANOVA using Tukey’s post-hoc comparisons (n=3). **(b)** Immunofluorescence staining for NF-κB p65 in Caco-2 cell monolayers treated with normoxia or hypoxia (6 or 12 hours). Scale bar = 50 μm. **(c)** ELISA for TNF-α, IL-1β and IL-8 in the supernatants of Caco-2 cell monolayers with (6 or 12 hours) or without hypoxia. Results of one-way ANOVA using Tukey’s post-hoc comparisons (n = 3). **(d)** Caco-2 cell monolayers were transfected with CFTR-silencing ribosome vectors (**rib**) or empty pEF/V5-His vectors as negative control (**pEF**). Representative Western blotting images and statistical analysis for P-ERK and ERK in Caco-2 cells transfected with pEF or rib vectors treated with normoxia or hypoxia; Results of unpaired *t*-test (n = 3). **(e)** Immunofluorescence staining of NF-κB p65 in Caco-2 cell monolayers transfected with pEF or rib vectors treated with normoxia or hypoxia. Scale bar =50 μm. Real-time quantitative PCR analysis **(f)** and ELISA **(g)** of TNF-α and IL-1β in the Caco-2 cell monolayers transfected with pEF or rib vectors, treated with an ERK inhibitor (**PD98059**), an NF-κB inhibitor (**Bay-11**) or a combination of both for 6 hours. Results of one-way ANOVA using Tukey’s post-hoc comparisons (n = 3). Western blotting **(h)** and statistical analysis **(i)** for CFTR, occludin and ZO-1 in Caco-2 cells transfected with pEF or rib in the presence (**plus sign**) or absence (**minus sign**) of an ERK inhibitor (PD98059), an NF-κB inhibitor (Bay-11) or a combination of both for 6 hours. **(j)** ELISA for TNF-α, IL-1β and IL-8 in the homogenates of ilea of DF508 +/+ and −/− mice. For +/+, n = 5; −/−, n = 3; Results of unpaired *t*-test. *p < 0.05, **p < 0.01, ***p < 0.001 and ****p < 0.0001. The experiments were repeated at least 3 times; results represent means ± SEM of 3 independent experiments performed in triplicate. *CFTR* cystic fibrosis transmembrane conductance regulator, *ERK* extracellular signal-regulated protein kinase, *ANOVA* analysis of variance, *ELISA* enzyme-linked immunosorbet assay, *P-ERK* Phospho-extracellular signal-regulated kinase, *TNF-α* tumor necrosis factor-α, *N* normoxi, *H* hypoxia, *IL-1β* Interleukin-1β, *IL-8* Interleukin-8, *NF-κB* nuclear factor-κB, *ZO-1* zonula occludens-1

Inflammation is known to damage intestinal epithelial junctional complexes. In addition, previous studies have demonstrated that inflammatory factors are modulated by CFTR. Therefore, we examined several pivotal inflammatory factors (TNF-α, IL-1β and IL-8) to explore whether they would play a role in the hypoxia-induced junctional disruption. As both NF-κB and MAPKs control the downstream transcription of inflammatory mediators and are known to be activated by hypoxia, their expression was also determined. As predicted, after hypoxia, both ERK and NF-kB were activated in Caco-2 cells, with maximum activation observed at 6 hours of hypoxia (Figures 3a, b). Increased release of TNF-α, IL-1β and IL-8 was consistently detected, with a peak after 6 hours of hypoxia treatment (Figure 3c). Moreover, knockdown of CFTR led to activation of ERK (Figure 3d) and NF-κB (Figure 3e); however, the combination of hypoxia and knockdown of CFTR failed to further activate ERK or NF-κB (Figures 3d and e), suggesting that hypoxia might modulate ERK and NF-κB through CFTR. Knockdown of CFTR led to an increase of TNF-α and IL-1β (Figures 3f, g) and a decrease of ZO-1 and occludin (Figures 3h, i), which were prohibited by either a specific ERK signaling inhibitor, PD98059, a specific NF-κB inhibitor, Bay 11-7082, or a combination of these. No activation of p38 MAPK or Jun N-terminal kinase was observed after knockdown of CFTR (data not shown).

In the *in vivo* experiment, higher levels of TNF-α, IL-1β and IL-8 were detected in the distal ileum of DF508 mutant (−/−) mice compared to DF508 wildtype mice (Figure 3j).

### Vitamin D3 reversed severe burn-induced EBT, possibly through CFTR/ERK/TNF-α/TJs

**Figure 4.**
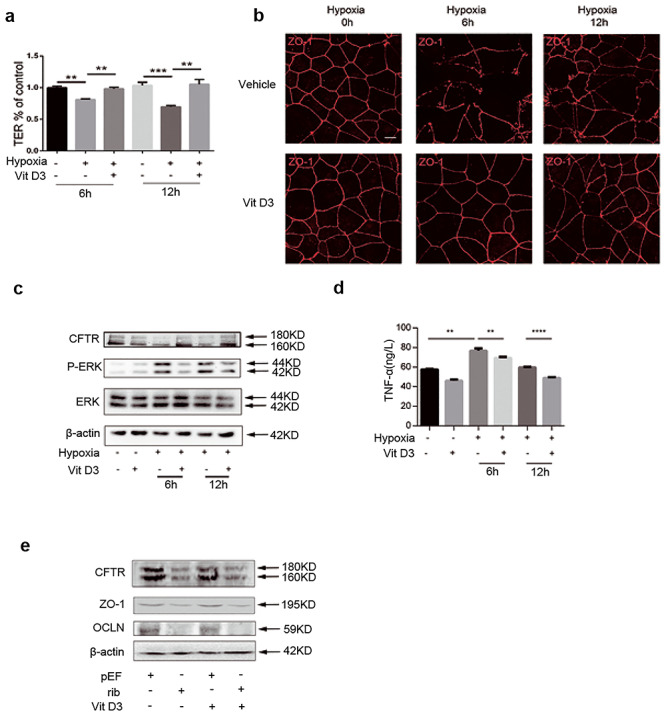
Vit D3 reversed ERK activation, TNF-α production and ZO-1 disruption induced by hypoxia. **(a)** TER assay of Caco-2 monolayers treated with hypoxia (**plus sign**) (6 or 12 hours) or normoxia (**minus sign**) in the presence (**plus sign**) or absence (**minus sign**) of vit D3. TER values were normalized to the control group. Repeated-measures analysis of variance was used to do the statistical analysis. Multivariate analysis of variance was used to compare the two groups at each time point. **(b)** Immunofluorescence staining for ZO-1 of Caco-2 monolayers underwent hypoxia (plus sign) (6 or 12 hours) or normoxia (minus sign) treatment in the presence (plus sign) or absence (minus sign) of vit D3. Scale bar = 50 μm. **(c)** Representative western blotting for CFTR, P-ERK and ERK of Caco-2 monolayers underwent hypoxia (plus sign) (6 or 12 hours) or normoxia (minus sign) treatment at the presence (plus sign) or absence (minus sign) of vit D3. **(d)** Enzyme-linked immunosorbent assay for TNF-α of Caco-2 monolayers treated with hypoxia (plus sign) (6 or 12 hours) or normoxia (minus sign) in the presence (plus sign) or absence (minus sign) of vit D3; n = 3. **(e)** Representative western blotting for CFTR, ZO-1 and occludin of control (**pEF**) and CFTR knockdown Caco-2 monolayers treated with or without vit D3. Results of one-way ANOVA using Tukey's post-hoc comparisons (n = 3). **p < 0.01, ***p < 0.001, ****p < 0.0001. The experiments were repeated at least 3 times. *ERK* extracellular signal-regulated protein kinase, *P-ERK* phospho-extracellular signal-regulated kinase, *TNF-α* tumor necrosis factor-α, *TER* transepithelial resistance, *CFTR,* cystic fibrosis transmembrane conductance regulator, *vit D3* vitamin D3, *ZO-1* zonula occludens-1

Previous studies have demonstrated that vitamin D3 prevents EBT via its anti-inflammatory and barrier-protective properties, but the underlying mechanism is unclear. In the present study, addition of vitamin D3 to Caco-2 monolayers reversed the decrease of TER induced by hypoxia (Figure 4a). Immunofluorescence staining for ZO-1 showed that hypoxia led to a discontinuous morphology of TJs, while treatment with vitamin D3 preserved ZO-1 labeling throughout (Figure 4b). Administration of vitamin D3 reversed the CFTR downregulation and ERK activation induced by hypoxia (Figure 4c). The increased TNF-α production induced by hypoxia was significantly suppressed by vitamin D3 (Figure 4d). Vitamin D3 failed to increase CFTR, ZO-1 and occludin expression in CFTR knockdown cells (Figure 4e).

**Figure 5.**
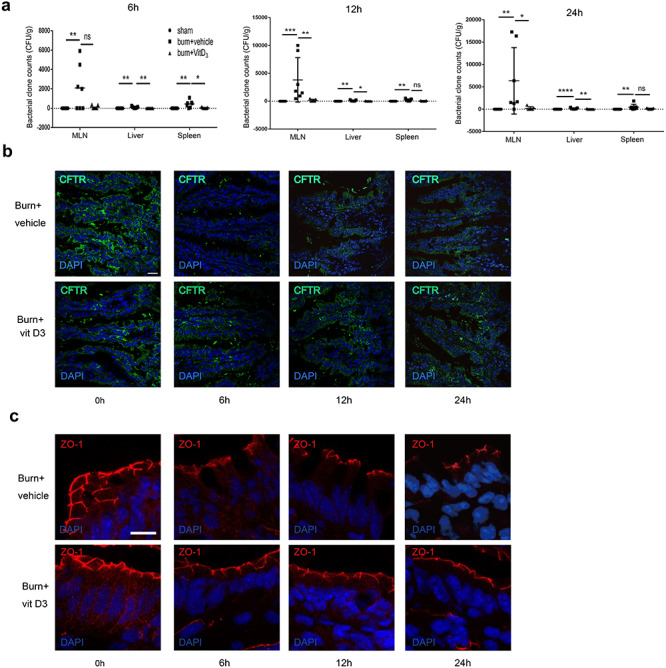
Vit D3 reversed severe burn-induced CFTR reduction, disruption of junctions and enteric bacterial translocation. **(a)** Statistical analysis of bacterial clone counts in lymph node, spleen and liver tissue homogenates with or without burn injury in the presence or absence of vit D3. Results of one-way ANOVA using Tukey's post-hoc comparisons (n = 7). **(b)** Immunofluorescence staining for CFTR of distal ileum in burnt mice treated with vit D3 or vehicle. Scale bar = 20 μm. **(c)** Immunofluorescence staining for ZO-1 of distal ileum in burnt mice treated with vit D3 or vehicle. Scale bar = 10 μm. All experiments were repeated at least twice. *CFTR* cystic fibrosis transmembrane conductance regulator, *DAPI* 4',6-Diamidino-2-phenylindole, *vit D3* vitamin D3, *ZO-1* zonula occludens-1

Bacterial count analysis of mesenteric lymph node, liver and spleen homogenates in sham, burnt and vitamin D3 pre-treated burnt mice showed that administration of vitamin D3 prevented severe burn-induced EBT (Figure 5a). Immunofluorescence staining suggested a time-dependent change in CFTR expression in the distal ileal epithelia of burnt mice, with a significant decrease at 6, 12 and 24 hours post burn, whereas vitamin D3 pre-treated burnt mice maintained a relatively high and steady CFTR level in the ileum (Figure 5b). Expectedly, coinciding with CFTR alteration, ZO-1 staining of the ileum showed a distinct reduction and mislocation in immunofluorescence at 6, 12 and 24 hours post burn injury, while treatment with vitamin D3 maintained a normal ZO-1 level and location in the intestinal epithelial barrier post burn (Figure 5c).

## Discussion

In this study, we examined one of the molecular mechanisms of intestinal barrier dysfunction and EBT post severe burn. Using an *in vitro* hypoxia model and a mouse 30% TBSA full-thickness dermal burn model, we have demonstrated that, under both conditions, CFTR in the intestinal epithelia is downregulated and that this is associated with a decline of crucial junctional proteins and, consequently, increased intestinal permeability. *In vitro*, knockdown of CFTR causes barrier dysfunction and increases production of critical cytokines, which is supported by parallel observations from the *in vivo* data on CFTR mutant mice. Furthermore, the *in vitro* data suggest that CFTR knockdown induces ERK and NF-κB activation. Administration of ERK or NF-κB inhibitors, or a combination of both, reverses the established barrier-disrupting and cytokine-promoting effects of CFTR knockdown. Finally, vitamin D3 reverses downregulation of CFTR in the intestinal epithelia, gut barrier disruption and EBT induced by severe burn, the mechanism of which may be via CFTR/ERK/TNF-α signaling.

It has been established that, after severe burn, the function of the intestinal epithelial barrier is compromised due to gut ischemia and hypoxemia, which occurs rapidly following the injury. A large number of studies indicate that EBT begins as early as a few hours post burn, peaks at 24 hours, and lasts as long as 9 days post burn [40–43]. Therefore, in the *in vivo* model, we chose to observe the key molecules and pathways at 6 and 24 hours post burn. A previous study has shown that though EBT peaks at 24 hours post burn, intestinal hypoxia does not last for 24 hours, since there was no significant difference in intestinal blood flow in sham and burnt rats at 24 hours post severe burn, while intestinal blood flow decreased significantly in burnt rats compared to sham rats at 5 hours post burn [44]. Hence, in our study, Caco-2 cells were treated with hypoxia for 6 and 12 hours.

We found decreased expression of ZO-1, occludin and E-cadherin and disorganized localization of ZO-1 in burnt mice compared to sham mice at 6 and 24 hours post burn. In agreement with our results, reduction and reorganization of ZO-1 and occludin were observed in the intestines of BALB/c mice that received a 30% TBSA steam burn in another study [43]. Interestingly, our study observed a change in CFTR coinciding with the alteration of ZO-1, occludin and E-cadherin.

Pro-inflammatory mediators produced by intestinal epithelia as a result of gut hypoxia are known to be negatively regulated by CFTR, causing gut barrier damage [45]. TNF-α and IL-1β are regarded as the major cytokines induced soon after burn injury. IL-8 is a particularly potent chemotactic factor for neutrophils and initiates the acute inflammatory cascade. These inflammatory mediators were identified as key players in the present study. Furthermore, we found that CFTR is a crucial link connecting enteric hypoxia and overproduced pro-inflammatory mediators in the mechanism of EBT post severe burn. After burn injury, the time-dependent alteration of CFTR expression is concomitant with a simultaneous change in junctional proteins. A reciprocal pattern of inflammatory factors is also detected. More importantly, either hypoxia or knockdown of CFTR leads to activation of ERK and NF-κB, contributing to excessive production of TNF-α, IL-1β and IL-8, and the disruption of TJs. In line with the *in vitro* results, greatly increased production of TNF-α, IL-1β and IL-8 and EBT are found in the distal ileum of ∆F508 mutant mice compared to their wild-type counterparts. Consistently, a previous study has reported disrupted TJs caused by elevated TNF-α in the small intestine of CF mice [22]. However, the intrinsic connection between CFTR and junctional complexes remains elusive. To the best of our knowledge, the present study has, for the first time, demonstrated the occurrence of EBT in F508 heterozygote (+/−) and mutant mice intestine and identified excessive inflammation leading to TJs breakdown as one of the mechanisms. Our study documents the increasingly complex role of CFTR in epithelial barrier function.

This evidence supports the notion that CFTR is an important molecule in the gut epithelia, and that defects of it result in a series of significant downstream events leading to EBT. Furthermore, the use of *in vivo* models supports the clinical significance of this work. Thus, therapies targeting CFTR may be beneficial in mitigating inflammation, protecting intestinal barrier integrity and preventing EBT following burn injury.

A previous study has shown that, after 12 or 24 hours of hypoxia, suppression of CFTR function was observed [46]. Our study has tested whether the effect of CFTR regulating TJs is dependent on its channel function by using a specific CFTR channel inhibitor, CFTRinh-172. Our results indicate that inhibition of CFTR by CFTRinh-172 has no significant effect on TER and TJ protein expression in Caco-2 monolayers (unpublished work by the current authors), suggesting that the role of CFTR in modulating TJs is independent of its channel function, which would be inconsistent with a previous study showing that TER was not affected by CFTRinh-172 in airway epithelial cells [21].

Apart from leaky TJs, enteric epithelial apoptosis also disrupts mucosal barrier integrity. CFTR has been shown to prevent apoptosis in a number of cells through the endoplasmic reticulum (ER) stress pathway [47–49]. In addition, excessive accumulation of pro-inflammatory cytokines due to decrease in CFTR induces apoptosis of intestinal epithelial cells [50]. Thus, reduced CFTR post severe burn may lead to apoptosis of gut epithelial cells, which may also contribute to EBT, though further data is needed to test this hypothesis.

Vitamin D is increasingly being recognized as having immunomodulatory and anti-inflammatory properties and a protective effect on the gut. A previous report has demonstrated that vitamin D deficiency is a common comorbidity in patients with CF and vitamin D is therefore routinely supplemented [51]. Our *in vitro* and *in vivo* data (Figures 4 and 5) show that vitamin D3 maintains CFTR levels and attenuates ERK activation, lowering TNF-α secretion and preserving normal function and morphology of TJs in the intestinal epithelia, and preventing EBT post severe burn. However, vitamin D3 fails to elevate CFTR, ZO-1 or occludin levels after knockdown by degrading CFTR mRNA, suggesting that a certain level of CFTR is necessary for vitamin D3 to exert a protective effect on TJs, and that the effects of vitamin D3 on maintaining CFTR level after hypoxic injury might be through post-transcriptional mechanisms rather than promotion of CFTR transcription. Intriguingly, another study has demonstrated that, when stimulated with IL-1β, vitamin D3 attenuates inflammation in CFTR knockdown intestinal epithelial cells but has no effect in cells with intact CFTR [52], possibly due to alterations in its catabolism associated with changes in CYP24A1 expression. Hence, the underlying relationship between vitamin D3 and CFTR still needs more exploration. Besides the mechanism of vitamin D3 protecting the gut barrier that the present study describes, the alternative hypotheses are that CFTR and vitamin D3 may improve hypoxic conditions in the intestine post severe burn; or that CFTR and vitamin D3 may inhibit enteric bacteria proliferation in extra-intestinal organs rather than purely modulating bacterial migration. These hypotheses await further investigation. However, the current findings have shed new light on the use of vitamin D3 in the prevention and treatment of EBT post severe burn.

The gastrointestinal tract plays a central role in initiating multiple organ dysfunction syndrome after surgical stress [53, 54]. EBT occurs early and sets in motion a series of severe systemic consequences, such as systemic inflammatory response syndrome, sepsis [55] and MOF. In addition, EBT is a ubiquitous pathophysiological process in critical patients with trauma, shock, burn injuries and other critical surgical illnesses. Hence, the molecular mechanism underlying EBT described in this study is of vital significance and has wide-ranging implications.

## Conclusions

The present study has clearly demonstrated that CFTR and downstream signaling are critical in modulating the intestinal epithelial junction and EBT post severe burn injury.

## Abbreviations

AJ: adherens junction; ANOVA: One-way analysis of variance; BSA: bovine serum albumin; CF: cystic fibrosis; CFTR: cystic fibrosis transmembrane conductance regulator; Ct: cycle threshold; DAPI: 4',6-Diamidino-2-phenylindole; DF508: CFTR gene mutation at position 508; EBT: enteric bacterial translocation; ELISA: Enzyme-linked immunosorbent assay; ERK: extracellular signal-regulated kinase; i.p. intraperitoneal; ER: endoplasmic reticulum; IL-1β: interleukin-1β; IL-8: interleukin-8; JNK: Jun N-terminal kinase; MAPK: mitogen-activated protein kinase; MLN: mesenteric lymph node; MODS: multiple organ dysfunction syndrome; NF-κB: nuclear factor-κB; PBS: phosphate-buffered saline; OCT:optimum cutting temperature compound; p-ERK: phospho-extracellular signal-regulated kinase; QRT-PCR: real-time quantitative PCR; TBSA: total body surface area; TER: transepithelial electrical resistance; TJ: tight junction; TNF-α: tumor necrosis factor-α; ZO-1: zonula occludens-1.

## Authors’ contributions

JC and YXZ designed the study. XZL, YC, BY, YP, YJC and ZCY performed the experiments. XZL and JC analysed the data. JC and YXZ drafted and revised the manuscript. All authors reviewed and approved the final version. JC and YXZ approved the final version for submission.

## Ethics approval

All procedures were approved by the Animal Ethical Committee of the Third Military Medical University and were carried out according to the approved guidelines of the animal center of Southwest Hospital, Third Military Medical University.

## Conflicts of interest

No relevant conflicts of interest
